# Role of surface energy and nano-roughness in the removal efficiency of bacterial contamination by nonwoven wipes from frequently touched surfaces

**DOI:** 10.1080/14686996.2017.1288543

**Published:** 2017-03-14

**Authors:** Nicholas W. M. Edwards, Emma L. Best, Simon D. Connell, Parikshit Goswami, Chris M. Carr, Mark H. Wilcox, Stephen J. Russell

**Affiliations:** ^a^ Nonwovens Research Group, School of Design, University of Leeds, Leeds, UK; ^b^ Department of Microbiology, Leeds Teaching Hospitals NHS Trust, Leeds, UK; ^c^ The Astbury Centre for Structural Molecular Biology, University of Leeds, Leeds, UK; ^d^ School of Physics and Astronomy, University of Leeds, Leeds, UK; ^e^ Fibre and Fabric Functionalisation Research Group, School of Design, University of Leeds, Leeds, UK; ^f^ School of Design, University of Leeds, Leeds, UK

**Keywords:** Nonwoven, pathogen, surface energy, nano-roughness, decontamination, medical, plasma, 60 New topics/Others, 212 Surface and interfaces, 600 Others: Medical

## Abstract

Healthcare associated infections (HCAIs) are responsible for substantial patient morbidity, mortality and economic cost. Infection control strategies for reducing rates of transmission include the use of nonwoven wipes to remove pathogenic bacteria from frequently touched surfaces. Wiping is a dynamic process that involves physicochemical mechanisms to detach and transfer bacteria to fibre surfaces within the wipe. The purpose of this study was to determine the extent to which systematic changes in fibre surface energy and nano-roughness influence removal of bacteria from an abiotic polymer surface in dry wiping conditions, without liquid detergents or disinfectants. Nonwoven wipe substrates composed of two commonly used fibre types, lyocell (cellulosic) and polypropylene, with different surface energies and nano-roughnesses, were manufactured using pilot-scale nonwoven facilities to produce samples of comparable structure and dimensional properties. The surface energy and nano-roughness of some lyocell substrates were further adjusted by either oxygen (O_2_) or hexafluoroethane (C_2_F_6_) gas plasma treatment. Static adpression wiping of an inoculated surface under dry conditions produced removal efficiencies of between 9.4% and 15.7%, with no significant difference (*p* < 0.05) in the relative removal efficiencies of *Escherichia coli*, *Staphylococcus aureus* or *Enterococcus faecalis*. However, dynamic wiping markedly increased peak wiping efficiencies to over 50%, with a minimum increase in removal efficiency of 12.5% and a maximum increase in removal efficiency of 37.9% (all significant at *p* < 0.05) compared with static wiping, depending on fibre type and bacterium. In dry, dynamic wiping conditions, nonwoven wipe substrates with a surface energy closest to that of the contaminated surface produced the highest *E. coli* removal efficiency, while the associated increase in fibre nano-roughness abrogated this trend with *S. aureus* and *E. faecalis*.

## Graphical abstract

**Figure:**
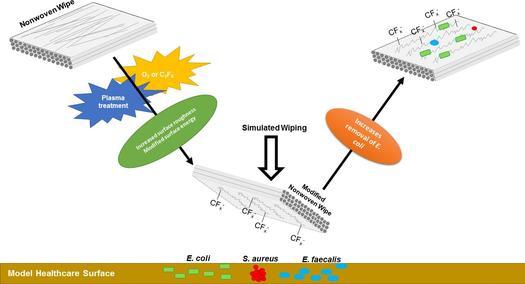

Plasma modification of the nano-roughness and surface energy of fibres in nonwoven wipes was found to influence the relative removal efficiencies of common bacterial pathogens from model healthcare surfaces under dynamic wiping conditions.

## 1. Introduction

Healthcare acquired infections (HCAIs) can be defined as any infection occurring within 48 h of hospital admission, three days of hospital discharge, or 30 days of an operation [1]. Such infections are a serious consequence of hospitalisation [2], causing both morbidity and mortality. In a recent assessment, HCAIs were directly associated with more than 37,000 deaths per annum in Europe [3].

Bacteria exist in either free-floating, planktonic or substratum-attached sessile states [4]. Surface attachment and subsequent biofilm formation is a key survival mechanism [5]. The process anchors the microorganism in an environment that is nutritionally advantageous [4], and provides increased resistance to chemical and physical insults [6,7]. Evidence from a large number of investigations, including studies modelling transmission routes [8], microbiologic studies [9], observational epidemiologic studies [10], intervention studies [11], and outbreak reports [12] has found that critical patient care surfaces contaminated with pathogenic bacteria contribute to the transmission of HCAIs. At least 20–30% of HCAIs are considered to be preventable by appropriate hygiene and control programmes [3], and so the effective removal of pathogens from surfaces in critical patient care areas is key. One currently employed strategy is to clean contaminated surfaces using nonwoven fabric wipes, either alone or in combination with detergents or biocides [13]. Whilst this is widely practised, the fundamental underlying interactions governing the removal of bacteria by the nonwoven fabrics remain poorly understood [14,15]. Considerable focus has understandably been placed on the role of the biocide or detergent used in combination with the wipe to decontaminate surfaces [16,17]. However, dry-wiping may have a role to play in achieving effective pathogen removal. Quick removal of bacteria from surfaces (and subsequent disposal of the contaminated fabric) without biocidal usage does not counteract the selection pressure surface colonising bacteria are exposed to, but can reduce total pathogen numbers on surfaces [18].

Bacterial adhesion to abiotic surfaces is known to be influenced by physicochemical and electrostatic interactions between the cell and surface [19]. Hydrophobicity is one of the key factors influencing this interaction [20], and the importance of surface nano-roughness in respect of adhesion has also been identified as an influential factor [21,22]. Surface wiping [23] and the removal of bacteria from solid surfaces by wipes has been investigated by Williams et al. [13] and Ramm et al. [24]. These studies have described reproducible methodologies for assessing wiping efficiency, but the focus was on the macro-scale removal of bacteria in the presence of detergent or biocide, rather than on the fundamental micro- or nano-scale interactions between the fibres in the wipe, bacteria and contaminated surface. It is of interest to decouple the effects of the detergent or biocide and the wipe fabric itself to understand the role that dry wiping might have in decontaminating solid surfaces.

Accordingly, our aim was to determine the role of fibre surface energy and surface roughness in removing bacteria from a model solid surface in the dry state without impregnation with a liquid biocide or detergent. In this way the basic design attributes of the wipe fabric can be explored in the context of bacterial decontamination. An inherently hydrophilic regenerated cellulose fibre (lyocell) and an inherently hydrophobic fibre, polypropylene (PP) were selected for as raw materials for wipe fabric production. Furthermore, samples of hydrophilic, high surface energy lyocell fabric were also functionalised by plasma exposure to: (a) reduce surface energy; and (b) increase the surface nano-roughness. These fabrics were then evaluated with fabrics composed of low surface energy, high surface nano-roughness PP fibres.

## 2. Materials and methods

### 2.1. Nonwoven production

To ensure satisfactory control of wipe substrate properties and enable reliable comparisons of wiping behaviour, fabric samples were prepared in-house, using pilot-scale nonwoven manufacturing processes at the University of Leeds. The manufacturing procedure was designed to replicate that commonly used to prepare wipes in an industrial context. Polypropylene fibre (T133 HY-Entangle, Fibervisions, Varde, Denmark) of 1.7 dtex linear density, 40 mm fibre length and lyocell fibre (Lenzing, Grimsby, UK – 1.7 dtex, 38 mm fibre length, dull) were mechanically pre-opened prior to carding. Parallel-laid webs of 60 g m^−2^ were manufactured using a 0.5 m wide worker-stripper card (Tatham Ltd, Rochdale, UK). Fabrics were then produced by hydroentangling the carded webs (Hydrolace) whilst supported on a woven support conveyor at specific energy of 3.47 MJ kg^−1^.

To ensure removal of residual fibre finish, all fabrics were scoured in a Roaches Rotohose rotary drum dyeing machine (Roaches, Birstall, UK) for 15 min at 60°C with 1 g dm^−3^ Hostapal NIN (Clariant Produkte GMBH; Frankfurt, Germany) and 2 g dm^−3^ sodium carbonate; using a liquor ratio of 20:1 [25]. Fabrics were then thoroughly rinsed and line-dried prior to further treatment.

### 2.2. Plasma functionalisation

To modify fibre surface properties, lyocell fabrics were exposed to hexafluoroethane (C_2_F_6_) or oxygen (O_2_) gas (BOC, Manchester, UK) in a PICO low temperature low pressure plasma coater (40 kHz; Diener GmbH, Ebhausen, Germany). Exposures were carried out at 150 W power, 12 cm³ min^−1^ gas flow rate. Exposure times were 30 s and 1, 2, 3, 4, 5, 10 or 20 min for C_2_F_6_ and 20 min for O_2_. Initial pressure in the chamber was 0.15 Torr. Post-exposure, samples were left to condition in a standard textile testing environment for at least 24 h before further analysis or testing.

### 2.3. Nonwoven surface energy

Surface energies of the nonwoven samples were evaluated using the Owens, Wendt, Rabel and Kaelble method [26]. Wetted length was calculated using n-hexane (Sigma Aldrich, Gillingham, UK), and contact angle values with ethanol, 1-octanol, cyclopentanol (all Sigma Aldrich) and distilled water. Experiments were performed using a KRÜSS K100 tensiometer (KRÜSS GmbH, Hamburg, Germany).

### 2.4. Fibre surface roughness

Fibre surface roughness was analysed via atomic force microscopy (AFM). A Dimension Fastscan atomic force microscope (Bruker, Billerica, MA, USA) was used in contact DC mode to probe the surface of lyocell; C_2_F_6_ treated lyocell (1 min, 4 min and 20 min treated samples); O_2_ treated lyocell (20 min); and PP under ambient conditions. Samples were mounted on a 10 mm diameter circular metal disc using epoxy resin. Nanoscope Analysis v1.5 software (Advanced Surface Microscopy, Inc., Indianapolis, IN, USA) was used to evaluate the resulting data.

### 2.5. Mass spectrometry

Time-of-flight secondary ion mass spectrometry (ToF-SIMS) (Intertek MSG, Wilton, UK) analysis of untreated lyocell and the 1, 4 and 20 min C_2_F_6_ plasma exposed lyocell nonwoven fabric was performed using an Ion-ToF-SIMS IV unit (ION-TOF GmbH; Münster, Germany) with a Bi^+^ source and an ion dose of less than 1 × 10^12^ ions cm^−2^. Both positive and negative ion spectra were acquired from a 200 μm × 200 μm area in the mass range *m/z* = 0–1500.

### 2.6. Bacterial strains

The microorganisms used in this study were *E. coli* (ATCC 25922), *S. aureus* (ATCC 29213) and *E. faecalis* (ATCC 29212) supplied by the Leeds Teaching Hospitals NHS Trust Microbiology department (LGI; Leeds, UK). Strains were cultured according to previously published methods: *E. coli* according to Hsu et al*.* [27], *S. aureus* according to Holinka et al*.* [28] and *E. faecalis* according to Gallardo-Moreno et al*.* [29].

### 2.7. Measurement of bacterial removal efficiency

Removal of bacteria from the contaminated surface was tested using methodology adapted from Williams et al*.* [13]. For brevity, only modifications to this protocol are described.

Alcohol-sterilised poly (methyl methacrylate) (PMMA) surface tiles (registered to ISO 9001) were inspected to ensure freedom from any defects. These were then inoculated with 20 μl bacterial cell culture suspended in phosphate buffered saline (PBS) with 0.3 g dm^−3^ bovine serum albumin (BSA). To simulate static wiping, a 20 mm diameter of the nonwoven fabric was pressed against the inoculated surface tile for 10 s with an applied force of 150 g. For dynamic wiping, a 900 mm^2^ section of the test fabric was attached to a 20 mm diameter boss, and fixed to a Caframo BDC2002 overhead stirrer (Caframo Limited, Ontario, Canada). This was rotated at 60 r min^−1^ for 10 s at 150 g ± 10 g applied force. Bacteria removal efficiency was calculated as in Equation 1:

(1) $$R=\left(C_{ct}-C_{wt}/C_{ct}\right)\times 100\;$$

where R = removal efficiency (%); *C*_*ct*_ = bacterial colonies recovered from control tile; and $C_{wt}$ = bacterial colonies recovered from wiped tile.

### 2.8. Surface wetting tension

The wetting behaviour of the PMMA test surface was measured with milli-Q water using an FTÅ 1000 contact angle goniometer (First Ten Ångstroms; Portsmouth, VA, USA). The tiles were tested either sterile, or following inoculation with 20 μl of either 0.015 g m^−2^ or 0.15.g m^−2^ BSA in PBS and subsequent air-drying.

### 2.9. Scanning electron microscopy

Scanning electron microscopy (SEM) was employed to image test surfaces and fabric samples before and after completion of each wiping test (section 0). Bacteria were fixed on the samples for 2 h in 2.5% glutaraldehyde, 0.1 M phosphate buffer, then washed twice in 0.1 M phosphate buffer for 30 min. Post-fixing was performed in 1% osmium tetroxide in 0.1 M phosphate buffer for 12 h. Samples were dehydrated using an ascending acetone series – 20, 40, 60, 80 and 100% for 30 min each. Samples were critical point dried using a Polaron E3000 critical point dryer (Quorum Technologies Ltd, Laughton, UK), with liquid carbon dioxide as the transition fluid. Samples were mounted on 13 mm pin stubs, which were coated with 5 nm platinum using a 208HR high resolution sputter coater (Cressington Scientific Instruments Ltd, Watford, UK). Samples were imaged using a Quanta 200F FEGESEM (FEI; Hillsboro, OR, USA) with an accelerating voltage of 3 kV, a working distance of 11.9 μm and a typical magnification of 20,000×.

### 2.10. Statistical analysis

All presented data are the results of at least three independent replicates. A one-way analysis of variance (ANOVA) at the 95% confidence interval with a *post hoc* Tukey’s test was performed, or a paired-sample *t*-test was conducted. All analyses were completed in MINITAB software, version 16 (Minitab Inc., State College, PA, USA).

## 3. Results and discussion

### 3.1. Plasma treatment

Plasma exposure of polymer surfaces is an established method of surface modification [30], and is particularly well documented as a method of adjusting surface energy, or the free energy per unit area (mJ m^−2^) [31] to influence physical phenomena such as wetting and adhesion between dissimilar materials. In the context of wiping efficiency, the adhesion between the bacteria and the PMMA surface tile as well with the fibres in the wipe is of interest since the relative forces will influence the nature of the attachment and reattachment behaviour.

**Figure 1.**
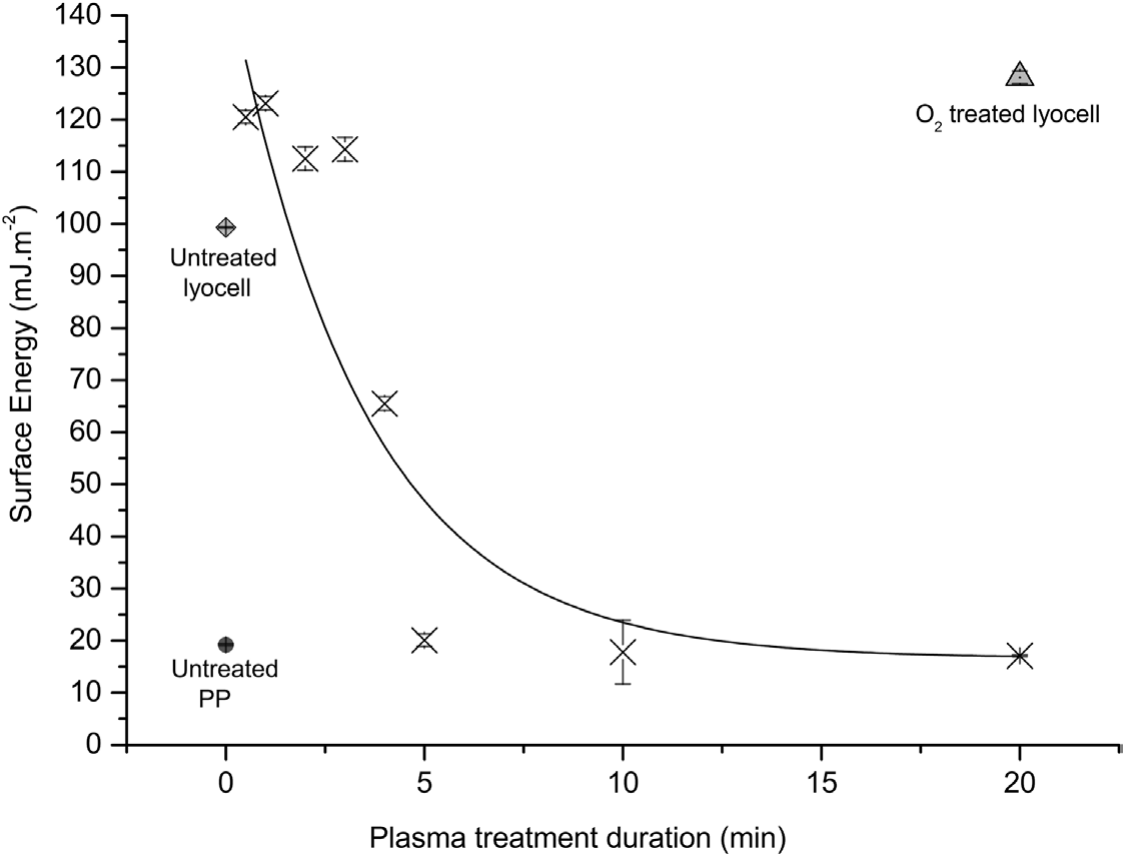
Surface energy of the nonwoven fabrics vs. plasma exposure time, measured via the OWRK method [26]. Fit line and data points for C_2_F_6_ treated lyocell, unless specified otherwise. Data are the mean of five replicates. Error bars = standard deviation.

By adjusting exposure time (Figure 1), lyocell fabric samples were produced with significantly different surface energies (*p* > 0.01) ranging from 128.1 mJ m^−2^ for a 20 min O_2_ exposure to 17.1 mJ m^−2^ for a 20 min C_2_F_6_ exposure, the latter value being close to that obtained for untreated PP fabric (19.2 mJ m^−2^), which is an inherently hydrophobic polymer. With lyocell samples treated with C_2_F_6_, an initial increase in surface energy was observed for exposure times of 30 s to 3 min, followed by a large decrease in surface energy for the 4 and 5 min exposed samples (Figure 1). This is a consequence of the increase surface roughness caused by the plasma exposure (Figure 3). Untreated lyocell is wettable, with a water contact angle of over 90°; therefore, an increase in the fibre surface roughness will increase both the wettability and the surface energy of the lyocell [32]. Longer plasma exposure times increase the nano-roughness of the fibre surface, as the sample is exposed for greater time in an energetic environment [33], It should be noted that plasma exposure alters both the surface energy and nano-roughness of a sample.

**Figure 2.**
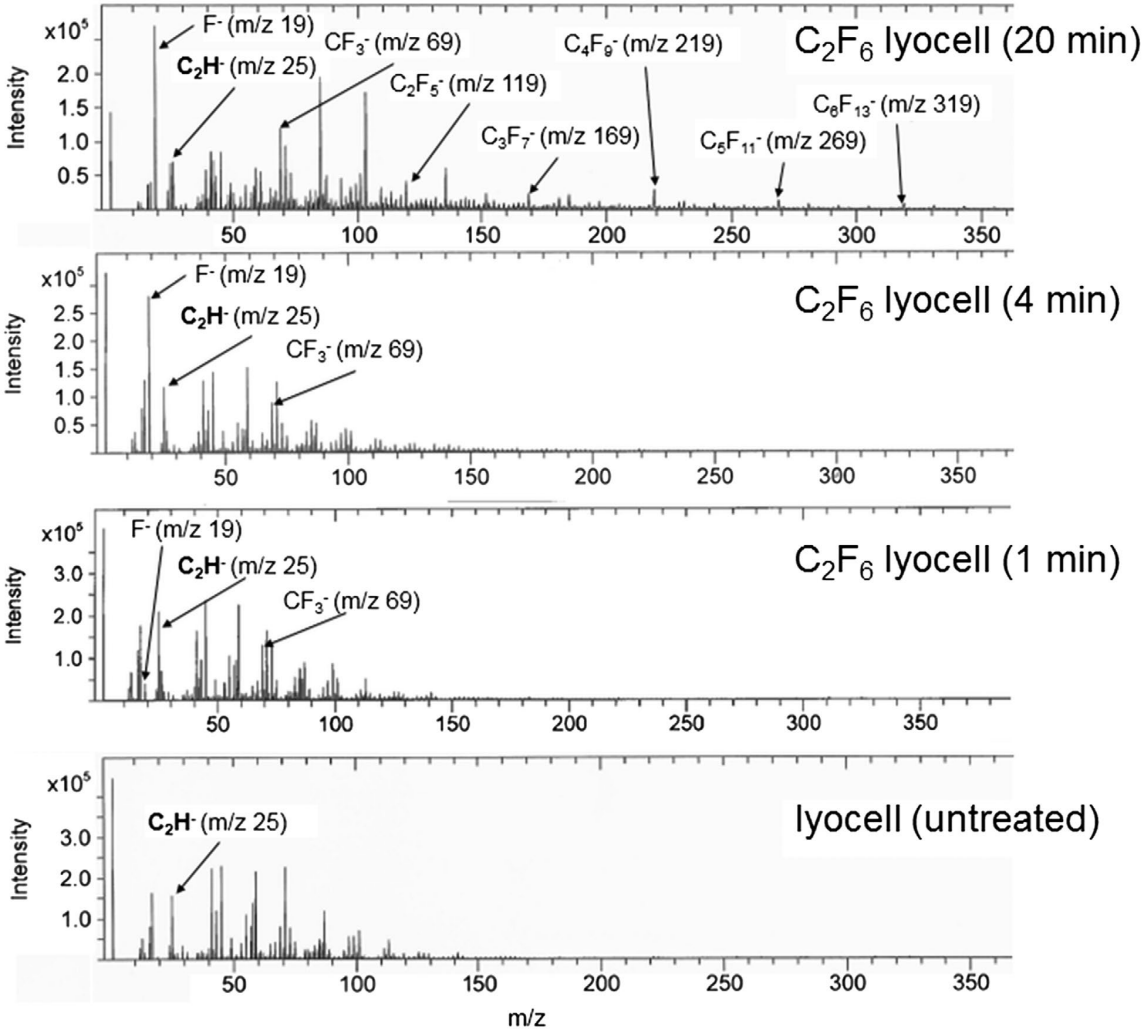
Negative ion ToF-SIMS spectra of untreated and C_2_F_6_ plasma treated lyocell nonwoven fabrics. Mass range m/z = 0–360.

**Figure 3.**
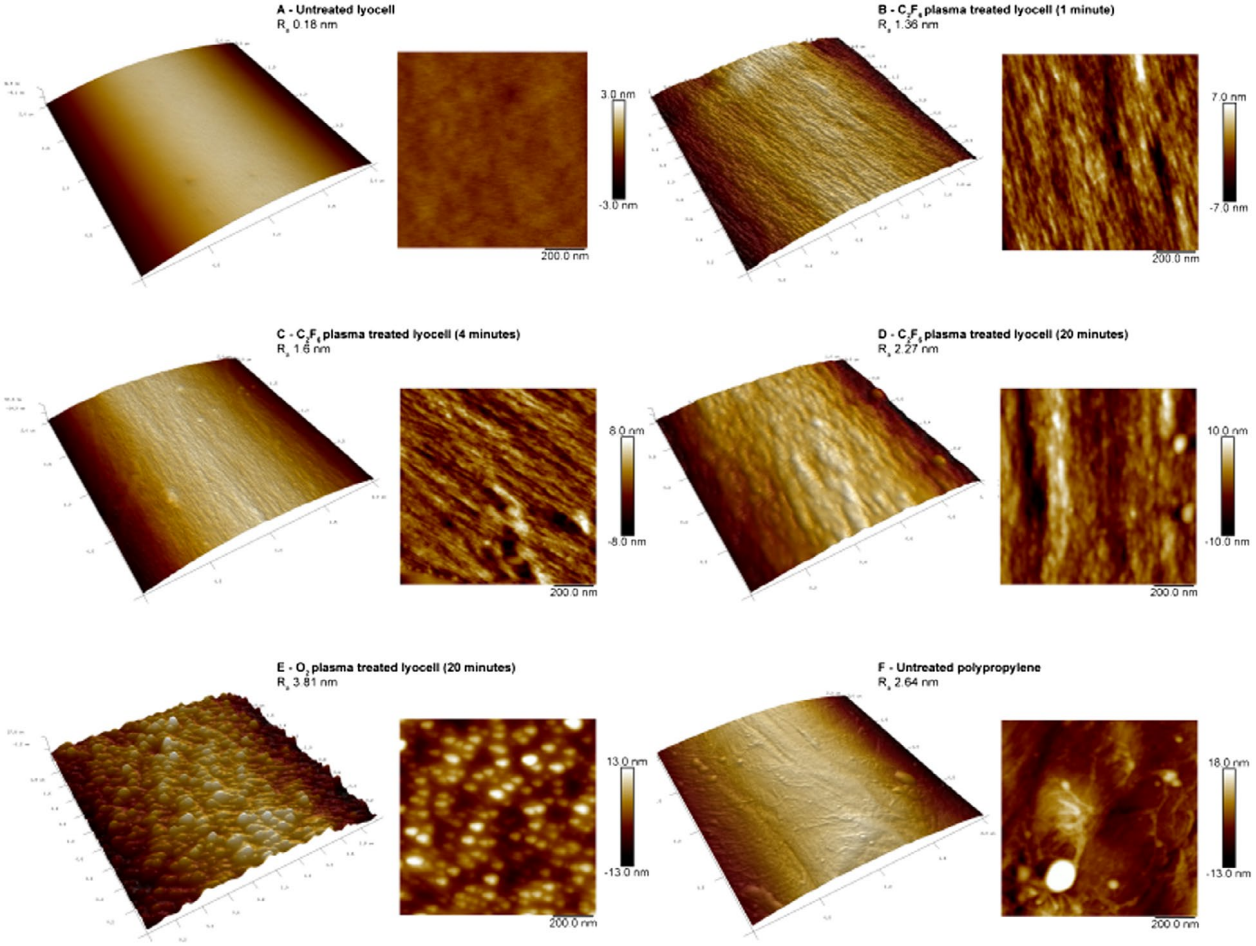
AFM micrographs of untreated and plasma treated fibre surfaces. (A) Untreated lyocell fibre surface; (B) C_2_F_6_ plasma-treated lyocell fibre surface after 1 min exposure; (C) C_2_F_6_ plasma-treated lyocell fibre surface after 4 min exposure; (D) C_2_F_6_ plasma-treated lyocell fibre surface after 20  min exposure; (E) O_2_ plasma-treated lyocell fibre surface after 20  min exposure; (F) untreated polypropylene fibre surface.

After a short exposure time (≤3 min), it is likely some surface chemical functionalisation occurs via oxidative fluorination, as confirmed by the ToF-SIMS spectra (Figure 2), resulting in an increase in surface polarity. However while gaseous fluorine is strongly electronegative and oxidative [34], with extended C_2_F_6_ plasma exposure an increase in F- or CF_x_ surface functionalisation can be expected to reduce surface energy. This is evident from the 4 min treated sample data onwards. There is also an increase in the nanoscale fibre surface roughness (Figure 3), associated with the reactive species in the plasma.

Note the greater influence of the O_2_ plasma exposure on the nanoscale fibre roughness compared with that obtained with C_2_F_6_ for the same exposure time. The effect is to increase surface energy in the lyocell samples, as a result of etching (Figures 2 and 3). [35]

Functionalisation of the fibre surfaces conferred by C_2_F_6_ plasma exposure was explored by means of the semi-quantitative ToF-SIMS technique. Examination of the negative ion spectra of all four C_2_F_6_ plasma exposed lyocell samples (Figure 2) indicates the presence of the C_2_H^–^ peak at m/z = 25, which can be used as an internal reference peak for monitoring surface compositional changes. Examination of the C_2_F_6_ plasma exposed lyocell samples indicates the surface F^–^ and CF_3_^–^ peak intensities increase with plasma exposure time. However during the low pressure RF plasma exposure the C_2_F_6_ gas is fragmented producing F^–^ and CF_X_^–^ radicals and these radicals in addition to reacting with the cellulosic surface can also polymerise on the cellulosic fibre surface. The presence of C_2_F_5_^–^, C_3_F_7_^–^, C_4_F_9_^–^, C_5_F_11_^–^ and C_6_F_13_^–^ on the 20 min plasma exposed lyocell ToF-SIMS spectrum confirms that significant plasma-enhanced chemical vapour deposition and polymerisation of CF_3_^–^ have occurred. The C_n_F_2n+1_ species are more abundant for lower *n* values. These results and the resulting increase in hydrophobicity at the fibre surface are consistent with the observed decreases in surface energy between the 1, 4 and 20 min exposure times.

The surface roughness of the nonwoven fabrics was measured via atomic force microscopy (AFM) (Figure 3). R_a_ is the arithmetic average of the roughness profile, and was calculated at 3 μm resolution. The untreated lyocell fibre (Figure 3(A)) shows a smooth, flat surface with no discernible nano-irregularities (R_a_ 0.18 nm). C_2_F_6_ plasma treatment of lyocell increased fibre surface roughness (Figure 3(B), (C) and (D)), progressively with increased plasma exposure time (R_a_ 1.36 nm for 1 min and 1.60 nm for a 4 min exposure time compared to R_a_ 2.27 nm for 20 min exposure time).

The surface morphology of the 1 min treated lyocell fibre was similar to that of the 4 min fibre. These surfaces revealed exposure of the underlying fibrillar structure of the fibre, but after extending the treatment time to 20 min, nodular structures were evident. This reflects the progressive modification of the fibre surface structure from the outside in during plasma exposure. In low pressure plasma exposure, surface etching occurs as fibres are bombarded with charged ions and electrons, subjecting their surfaces to a physical sputtering effect alongside chemical effects [36]. The sputtering can lead to micro- or nano-roughness on the fibre surfaces, exposing the underlying structure by removing surface material as a result of the energy input during the plasma exposure process [37]. Low temperature plasma techniques are surface selective in this regard [38].

Etching is more commonly observed with non-polymerising gases such as O_2_ than with depositing gases such as C_2_F_6_ [39], hence the higher surface roughness observed in lyocell fibres treated for 20 min in O_2_ compared with the same period in C_2_F_6_ (Figure 3(E) and (D)). In the former, many irregularities with nodular structures and substantial roughness (R_a_ 3.81 nm) were observed compared to the unexposed control (R_a_ 0.18 nm). This has been observed by Kale and Desai [40] and is attributed to plasma etching. In contrast, the 1, 4 and 20 min C_2_F_6_ exposed lyocell samples exhibited fibre surfaces resembling tree bark, with micro-fissures being evident, whereas the O_2_ plasma exposed lyocell sample exhibited a more granular morphology. The untreated PP fibres (Figure 3(F)) exhibited randomly distributed irregularities, as a result of the fibre manufacturing process, with a R_a_ value of 2.64 nm.

Plasma exposure therefore results in modification of fibre surface morphology via physical etching, introducing nano-roughness, as well as chemical modification. Both can modify surface wetting, and therefore adhesion behaviour. Initially, the surface roughness increased as a direct result of fibre etching in the RF plasma_;_ evidenced by the increase in surface energy from the 30 s to 3 min exposure time (Figure 1). The chemical functionalisation necessary to reduce surface energy (with CFx-) does not occur quickly and the polymerisation rate is dependent on the chemical activity of the plasma [41]. As expected, the lyocell fibre surfaces subjected to the longest C_2_F_6_ exposure time exhibited the highest degree of functionalisation and the lowest surface energy. The differences in fibre surface morphology (i.e. increase in surface nano-roughness after the same treatment time) between the C_2_F_6_ and O_2_ exposed surfaces after 20 min exposures are due to the gasses used with the C_2_F_6_ plasma providing a polymerisable deposition while the O_2_ plasma is a non-depositing surface modification. Longer plasma exposure times also increased the nano-roughness of the fibre surface [33].

### 3.2. Wetting behaviour of the wiping surface

The water contact angle on the wiping surface can be expected to change according to the bacterial contamination placed upon it and was measured before and after the surface was contaminated with the simulated organic load. An increase in the organic load increased the water contact angle and decreased the wetting tension (Table 1). Light and heavy organic loads have been previously simulated in wiping studies using 0.015 g m^−2^ BSA and 0.15 g m^−2^ BSA respectively [13,16]. In this study light organic load conditions were adopted.

**Table 1.** Mean contact angles and wetting tensions of PMMA wiping surfaces according to organic load, as measured by goniometry.

Organic load	Water contact angle	Wetting tension
Unsoiled surface(alcohol sterilised)	29.2°	63.5 mJ m^−2^
Low organic load (0.015 g m^−2^ BSA)	62.3°	33.8 mJ m^−2^
High organic load(0.15 g m^−2^ BSA)	81.4°	10.9 mJ m^−2^

Table 1 indicates an increase in contact angle and a decrease in wetting tension as the level of organic load increased. While this is expected given the chemical nature of BSA (protein), the salts in the PBS will also deposit on the surface, leading to an increase in surface roughness [42], increasing the contact angle and decreasing the wetting tension of the PMMA surface.

### 3.3. Bacterial removal from solid surfaces

Wiping experiments using nonwoven substrates manufactured in house, using industrially applicable nonwoven manufacturing processes, were undertaken to determine the influence of nano-roughness and fibre surface energy on the bacterial removal using the ‘low organic load’ conditions outlined in Table 1.

#### 3.3.1. Antibacterial activity

The agar diffusion plate test can be used to determine the effect of antibacterial agents applied to textiles [43]. In this instance it was used to assess whether the plasma functionalisation of the fibres in the nonwoven substrates led to a biocidal effect. The PP, unexposed lyocell, 20 min C_2_F_6_ exposed lyocell and 20 min O_2_ exposed lyocell all demonstrated ‘insufficient’ antibacterial effects against *E. coli*, *S. aureus* and *E. faecalis* according to the guidelines of the ISO 20645 method [44] (data not shown). Therefore, none of the fabrics were inherently biocidal before or after plasma exposure, based on this particular assay and relatively short timescale.

#### 3.3.2. Removal of bacteria via adpression

**Figure 4.**
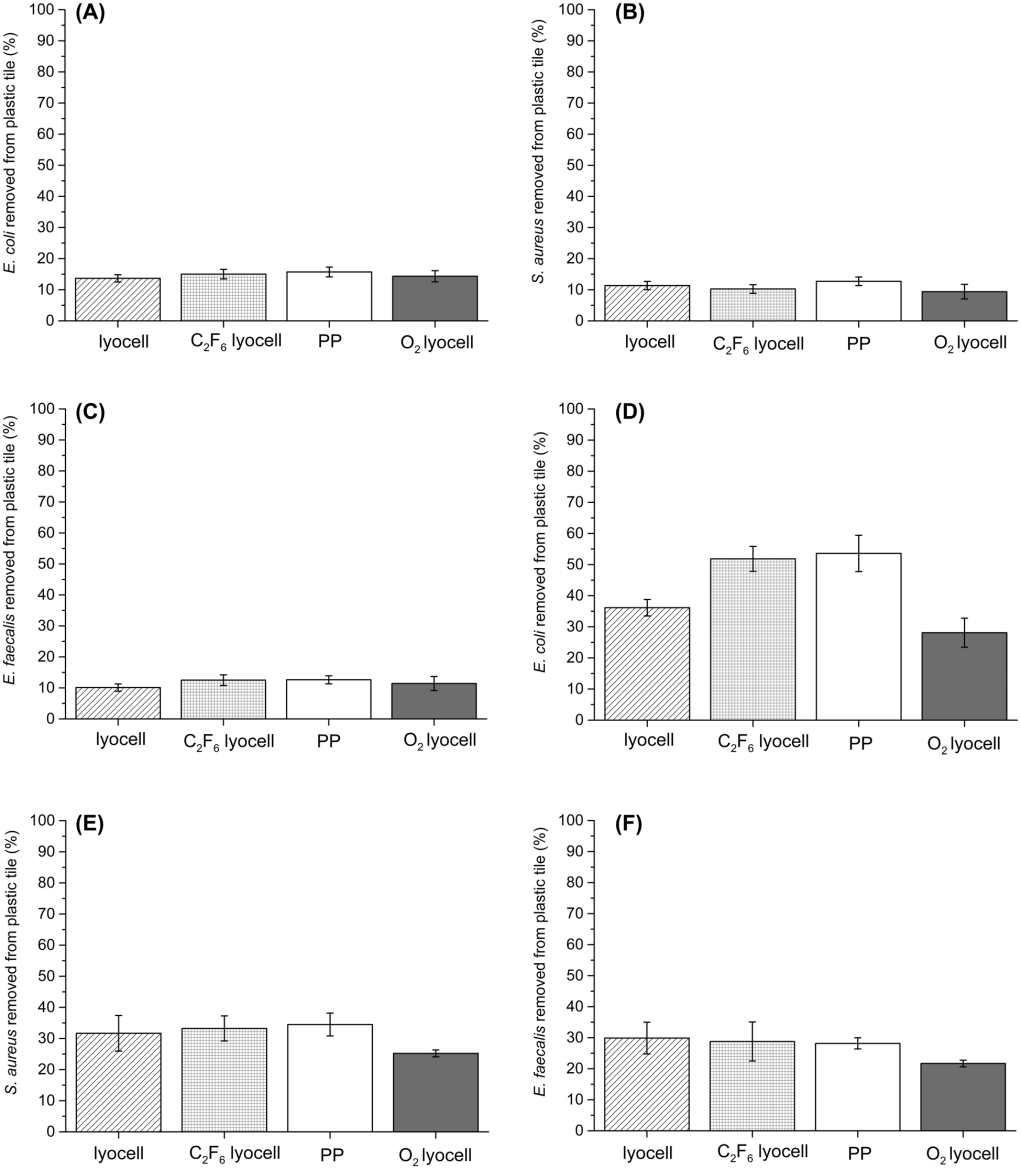
Bacterial removal by wiping. (A) Mean *E. coli* removal by adpression method; (B) mean *S. aureus* removal by adpression method; (C) mean *E. faecalis* removal by adpression method; (D) mean *E. coli* removal by dynamic wiping; (E) mean *S. aureus* removal by dynamic wiping; (F) mean *E. faecalis* removal by dynamic wiping. Data presented are the average of nine replicates and error bars represent standard error of the mean.

To decouple the influence of the physical wiping mechanism from direct interaction between the contaminated surface and the fibres in the wipe, a static adpression experiment was performed. All removal values were low with none exceeding 16%. There was no significant difference between the four wipes in terms of *E. coli* (Figure 4(A)), *S. aureus* (Figure 4(B)) or *E. faecalis* (Figure 4(C)) removal efficiency. The removal efficiencies for *E. coli* were generally higher than those for *S. aureus* and *E. faecalis* (*p* > 0.01). This may be due to the *E. coli* surface appendages (observed during dynamic wiping, Figure 5(A–D)) allowing the bacteria to overcome unfavourable surface topographies and better adhere to fibres under adpression conditions than either *S. aureus* or *E. faecalis* could. As in dynamic wiping, adpression involves physical contact between the face side of the wipe with the bacterial contamination residing on the contaminated surface; however, in the presence of applied pressure alone, the force of adhesion between the fibres and bacteria are insufficient to overcome resisting forces within the bacterial contamination itself or with the model wipe surface. Consequently, substantial bacterial transfer to the fibres in the wipe surface is inhibited.

Figure 5. SEM micrographs of *E. coli* (A–D), *S. aureus* (E–H) and *E. faecalis* (I–L) on unexposed lyocell fibres (A, E, I), 20 min C_2_F_6_ exposed lyocell fibres (B, F, J), 20 min O_2_ exposed lyocell fibres (C, G, K) and PP fibres (D, H, L) within each nonwoven fabric sample after dynamic wiping.

**Figure:**
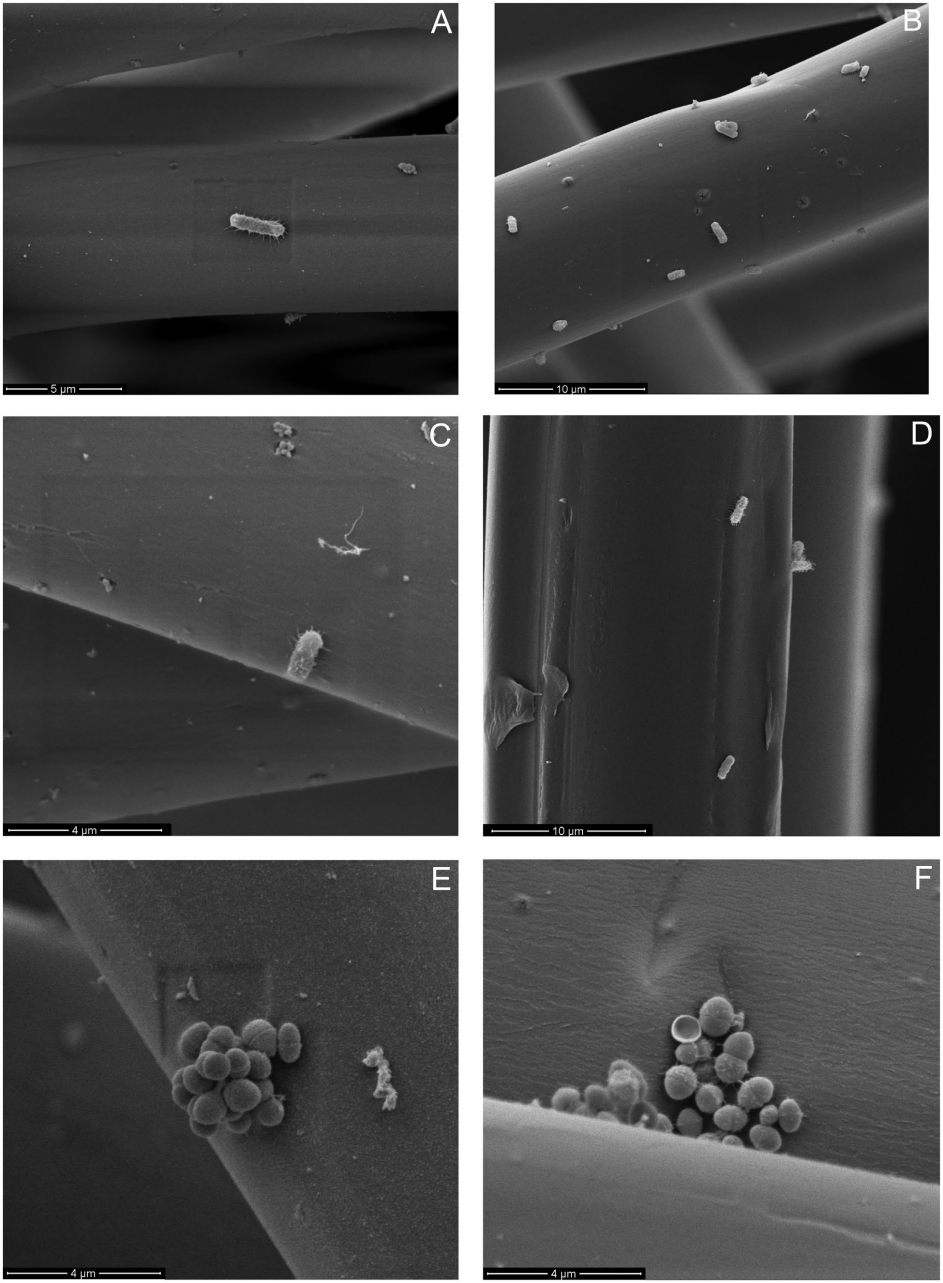

**Figure:**
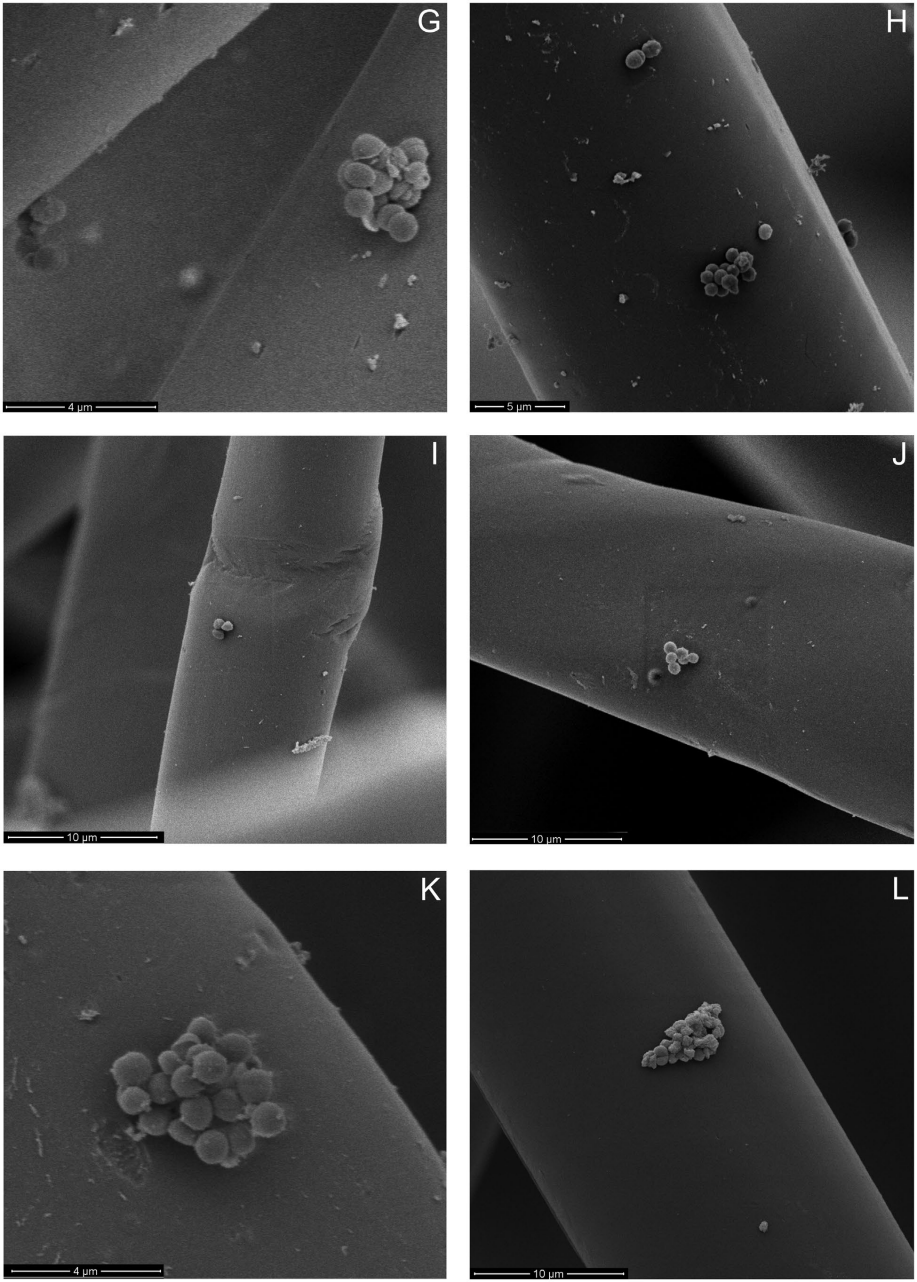

#### 3.3.3. Dynamic wiping

Bacteria will adhere more preferably to a surface which is of a surface energy closest to their own, i.e. hydrophobic bacteria will adhere better to hydrophobic surfaces and hydrophilic bacteria to more hydrophilic surfaces [20]. Therefore, it may be hypothesised that a reduction in surface energy of a lyocell fibre to a value closer to that of a surface soiled with a mixture of bacteria and proteins (the simulated organic load) will increase the bacterial removal efficiency of the lyocell fabric during wiping. It is anticipated the 20 min C_2_F_6_ treated lyocell and PP samples have surface energies closest to the wetting tension value of the soiled PMMA surface used in the wiping experiments and the control and O_2_ functionalised lyocell will have much greater surface energies than the wetting tension of the PMMA surface, so can be considered less favourable surfaces for bacterial adhesion.

The dynamic wiping data revealed markedly higher removal efficiencies than were achieved under adpression conditions. The PP and 20 min C_2_F_6_ exposed lyocell samples did remove significantly more *E. coli* (Figure 4(C)) than the other substrates, while there was no significant difference in removal between the different substrates for either *S. aureus* (Figure 4(E)) or *E. faecalis* (Figure 4(F)) (all ANOVA, *post hoc* Tukey’s test, *p* < 0.05). As expected, the O_2_ treated lyocell fabrics exhibited the lowest removal efficiency for all bacteria. The hydrophobic PP and the 20 min C_2_F_6_ exposed lyocell wipes exhibited significantly greater removal efficiency of *E. coli* (ANOVA, *post hoc* Tukey’s test *p* < 0.01).

These findings can be considered in terms of: The bacteria present in the simulated soil are in an environment that favours adhesion to surfaces of a similar surface energy. Essentially, the relative force of adhesion between bacteria and the contaminated surface on which they reside influences the force that is required to facilitate removal and adhesion to the fibres in the wipe during wiping [45]. In contrast to adpressure conditions, in dynamic wiping shear as well as compressive forces are applied, which will assist transfer to the fibre surfaces, overcoming the adhesive forces between bacteria and surface [45]. This removal threshold is less likely to be reached during the adpression experiments, as evidenced by the lower bacterial removal values.

Given their ‘favourable’ surface energies, bacterial adhesion to the PP and 20 min C_2_F_6_ lyocell fabrics was expected to be greater than to the O_2_ treated lyocell or unexposed lyocell substrates. However, both the PP and 20 min C_2_F_6_ lyocell wipe fibres, in addition to lower surface energy, also have increased surface nano-roughness compared with the unexposed lyocell. This additional factor is important because the thin and fluid outer membrane of Gram-negative bacteria such as *E. coli* will adhere better to the wipe’s increased fibre nano-roughness, allowing greater contact between the surface of the bacteria and the wipe, leading to an improved removal of the bacterial cells from the abiotic surface. This is indeed reflected in the observed increased *E. coli* removal efficiency values for the PP and 20 min C_2_F_6_ lyocell wipes.

In contrast, owing to their thicker peptidoglycan outer membranes and reduced conformation to the roughened fibre surfaces, no significant difference was observed between the removal efficiencies of the two Gram-positive bacteria (*S. aureus* and *E. faecalis*) under dynamic wiping conditions. The relatively low bacterial removal efficiency of the unexposed lyocell and the O_2_ exposed lyocell substrate during the dynamic wiping experiments can also be attributed to their unfavourable high surface energy values relative to the wiping surface (Figure 1). Although both surface energy and nano-roughness change with plasma treatment, the Gram-negative *E. coli* can better adapt to the unfavourable surface roughness than the Gram-positive *S. aureus* and *E. faecalis.* Therefore, for the favourable surface energy/unfavourable surface roughness (for bacterial adhesion and removal) PP and C_2_F_6_ treated lyocell nonwovens, more *E. coli* is removed than *S. aureus* or *E. faecalis*. The untreated lyocell has unfavourable surface energy, while the O_2_ treated lyocell nonwoven has both unfavourable surface energy and unfavourable surface roughness.

Substantial differences were observed between static (adpression) and dynamic wiping (Table 2). Based on a paired-sample *t*-test at the 95% confidence interval, dynamic wiping removed significantly more bacteria than static wiping irrespective of fabric treatment and the resulting surface energy. This is to be expected, as greater energy input is associated with dynamic wiping [46], and the applied forces involve shear as well as compression. The results confirm that dry wiping without the use of biocidal lotion or detergent can still remove bacteria from contaminated surfaces, supporting the conclusions of Wren et al*.* [47] and Koh et al*.* [23].

**Table 2.** Comparison of static wiping vs. dynamic wiping via paired-sample *t*-test.

	Wipe sample	Significant difference	*p*-value
*E. coli*	Lyocell	+++	<0.001
	C_2_F_6_ lyocell (20 min)	+++	<0.001
	O_2_ lyocell (20 min)	+	0.002
	PP	+++	<0.001
*S. aureus*	Lyocell	+	0.008
	C_2_F_6_ lyocell (20 min)	++	0.005
	O_2_ lyocell (20 min)	+++	<0.001
	PP	+++	<0.001
*E. faecalis*	Lyocell	+	0.025
	C_2_F_6_ lyocell (20 min)	+	0.009
	O_2_ lyocell (20 min)	+++	<0.001
	PP	+	0.001

The observed changes in surface energy and nano-roughness in lyocell fibres resulting from plasma exposure will influence bacterial adhesion [22,48]. In Gram-positive bacteria, surface roughness has the ability to limit the number of anchoring points, reducing the surface area in contact with the membrane, which impairs adhesion. In contrast, the outer membrane in Gram-negative bacteria is both more fluid and thinner than the outer peptidoglycan layer of Gram-positive bacteria [49]. Previous adhesion studies indicate that increasing the nano-roughness of a steel surface can significantly decrease the attachment of *S. aureus*, but has no effect on the adhesion of *E. coli*.[21] Nano-patterning on cicada wings has been found to be biocidal to Gram-negative bacteria, but not to Gram-positive bacteria adsorbed onto their surfaces, because of differences in cell membrane thickness [50]. One important distinction is that in these previous studies, data were obtained over 24 h and involved no dynamic wiping mechanism. By contrast, in the present work the contact time is only 10 s at a 60 r min^−1^ wiping rotation speed. This may explain why there was no evidence of nano-roughness induced biocidal activity, with differences between samples being confined to the bacterial removal efficiency.

The bacterial cell suspension solution was adjusted to the McFarland standard 0.5 – equivalent to an approximate cell density of 1 × 10^8^ CFU ml^−1^ [51], indicating there would be 2 × 10^6^ cells in the 20 μl of suspension inoculated onto the PMMA tile. This is likely to be artificially high [52], though necessary for bacteria quantification. This work is of significance to HCAI research as this studies the *in vitro* impact of physical wiping alone where at present there is little reported information.

### 3.4. Interaction of bacteria with wipe fibre surfaces under dynamic wiping conditions

It is evident in Figure 5 that the bacteria are collected mainly on fibre surfaces, rather than between the fibres themselves. Agglomerations of bacteria were randomly distributed along the fibre surfaces, and regions in which a single bacterium was present were also in evidence. No notable variations were observed for different bacteria in terms of their distribution along individual fibres after wiping, irrespective of the fabric type, i.e. in terms of fibre composition or plasma exposure. *E. coli* tended to adhere as a single bacterium; while *S. aureus* and *E. faecalis* tended to adhere in colonies. *E. coli* surface appendages can be observed in Figure 5(A–D), which is indicative of flagella acting as structural elements, enabling the bacterium to overcome unfavourable surface topographies [53]. This further explains the relatively high removal efficiency of *E. coli* removal by the hydrophobic PP and 20 min C_2_F_6_ exposed lyocell wipe fabrics.

Gram-negative bacteria are known to facilitate adhesion through pili or fimbriae [54,55], but, as expected, no appendages from adhered *S. aureus* were observed (Figure 5(E–H)) [56]. Pili were observed with *E. faecalis* (Figure 5(I–L)) and these appendages are known to contribute to adhesion and biofilm formation, particularly in endocarditis and urinary tract infections [57]. Only the fibres present at the surface of the wipe were imaged, so no conclusions can be drawn regarding bacterial penetration into the wipe.

## 4. Conclusions

Removal of pathogenic bacteria from abiotic surfaces using nonwoven wipes is a stratagem commonly used by healthcare providers to reduce the extent of bacterial contamination. The relative surface energies of the wipe fibres and the contact surface influence dry wiping efficiency, as well as the surface roughness of the fibre, but the impact depends on the type of bacterium. Reduction of the surface energy of lyocell nonwoven fibres by C_2_F_6_ plasma exposure increased the removal efficiency of *E. coli*, but there was no significant effect on the elimination of Gram-positive bacteria (*S. aureus* and *E. faecalis*). This suggests that modification of the surface energy of a nonwoven can increase the removal of bacteria, providing that the bacterial cell membrane conformation can adapt to the changes in surface nano-roughness caused by the plasma treatment. Dynamic wiping removes significantly more bacteria than adpression for all bacteria and wipe types. Bacteria are shown to adhere to fibres in a wipe during a wiping process without either biocide or detergent.

## Disclosure statement

No potential conflict of interest was reported by the authors.
